# Clinicopathological and prognostic implications of ALK rearrangement in patients with completely surgically resected lung adenocarcinoma

**DOI:** 10.1111/1759-7714.14170

**Published:** 2021-10-01

**Authors:** Huan Zhang, Guangyao Shan, Benjie Cai, Guoshu Bi, Zhencong Chen, Jiaqi Liang, Valeria Besskaya, Yuansheng Zheng, Weigang Guo, Lin Wang, Songtao Xu, Cheng Zhan

**Affiliations:** ^1^ Department of Thoracic Surgery Zhongshan Hospital, Fudan University Shanghai China; ^2^ Department of Thoracic Surgery and Urology Shigatse People's Hospital Tibet Autonomous Region China

**Keywords:** ALK inhibitors, anaplastic lymphoma kinase (ALK), clinical feature, lung adenocarcinoma, prognosis

## Abstract

**Background:**

The prognostic significance of ALK rearrangement is still contradictory. Here, we aimed to investigate the clinical characteristics and outcomes of lung adenocarcinoma patients with ALK rearrangement, and analyze whether these patients benefited from targeted therapy.

**Methods:**

This was a retrospective cohort study of 80 ALK‐rearranged lung adenocarcinoma patients who had undergone radical surgery and another 3031 ALK mutation‐negative patients were retrospectively reviewed for inclusion in this case‐controlled analyses. Overall survival (OS) was evaluated using the Kaplan‐–Meier method. Univariate analysis (UVA) and multivariate analysis (MVA) by the Cox proportional hazards regression identified risk factors that predicted OS.

**Results:**

Compared to ALK‐negative patients, the ALK rearranged patients were younger, with more non‐smokers, more females, a larger primary tumor was demonstrated, and were a higher pathological stage. In particular, the risk of lymph node metastasis was higher. For patients with surgically‐resected tumors, the prognosis was better for ALK rearranged patients (HR = 0.503; 95% CI: 0.259–0.974, *p* = 0.041). In addition, for stage II–III patients, targeted therapy was an independent prognostic factor of better OS (HR = 0.159; 95% CI: 0.032–0.801, *p* = 0.026).

**Conclusions:**

ALK rearranged lung adenocarcinoma patients who have undergone radical surgery have distinct clinical features. Patients with ALK rearrangement may have a favorable prognosis, and stage II–III patients may benefit from targeted treatment.

## Graphical abstract

The article compared 80 ALK+ and 3031 ALK‐ lung adenocarcinoma patients after surgery, explored the clinicopathological characteristics and prognostic features of ALK+ lung adenocarcinoma patients, and analyzed the impact of targeted drugs on the survival of ALK+ lung adenocarcinoma patients after surgery.

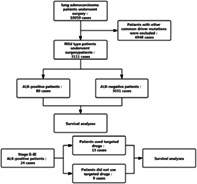

## INTRODUCTION

Lung cancer is the foremost cause of cancer death, and its incidence was also reported to be the second‐highest in 2020,^1^ which brings a great burden to society and the economy.^2^ Adenocarcinoma is the most common histological subtype.^3^

Genetic mutations often occur in patients diagnosed with lung adenocarcinoma. The most common genetic alterations are epidermal growth factor receptor (EGFR) and V‐Ki‐ras2 Kirsten rat sarcoma viral oncogene homolog (KRAS) activating mutations.^4^ Additional relatively rare genetic alterations in lung adenocarcinoma, such as ALK, have also attracted attention in recent years. ALK rearrangement is clinically important. Previous studies have revealed that the clinical characteristics of ALK‐positive lung adenocarcinoma are unique,^5^ including younger, more non‐smokers, more patients with adenocarcinoma, and more female patients. In addition, *ALK* gene mutations also have reference significance for prognosis. To date, the clinicopathological and prognostic features of ALK rearranged patients have not been investigated fully due to their relatively low incidence. Our study followed up and reviewed the prognosis and clinical characteristics of 80 ALK rearranged lung adenocarcinoma patients and 3031 ALK‐negative patients. The aim of the study was to provide a reference for further research on the studies of ALK rearranged lung adenocarcinoma and verify whether after complete resection these patients can benefit from targeted drugs.

## METHODS

### Patient selection

All ALK‐positive lung adenocarcinoma patients who had undergone surgery at Zhongshan Hospital, Fudan University, between 2016 and 2019 were retrospectively analyzed. Exclusion criteria were patients with squamous cell carcinoma, benign tumors, tumors with other common driver mutations (*EGFR*, *KRAS*, *HER2*, *ROS1*, *RET*, *BRAF*, *PIK3CA* and *NRAS*) or other tumors other than ALK‐positive lung adenocarcinoma. A total of 80 patients between September 2016 and October 2019 were finally included in the study. A total of 3031 wild‐type patients (without *EGFR*, *KRAS*, *HER2*, *ROS1*, *RET*, *BRAF*, *PIK3CA* and *NRAS* mutation) who received radical surgery between September 2016 and October 2019 were retrospectively reviewed for inclusion in this case‐controlled analyses. In addition, 303 wild‐type patients were selected for survival analyses by 1:10 random sampling.

All operations were carried out by thoracic surgeons in Zhongshan Hospital, and resected tumors and lymph nodes were reviewed by two experienced pathologists.

We collected postoperative data through outpatient follow‐up and annual telephone follow‐up. The duration of follow‐up for ALK‐positive patients was 12 to 53 months (median, 38 months) and for ALK‐negative patients was 12 to 55 months (median, 38 months); the last follow‐up date was November 2020. Patients were censored at last follow‐up if the patient was still alive or lost to follow‐up. Patients who died from noncancer cause were censored at the time of their death. Patients were excluded if their vital status or follow‐up times was unknown.

### Clinicopathological characteristics

The survey collected the following demographic and clinical data: (1) patient information: age at diagnosis, sex and smoking history; (2) tumor information: tumor size, primary location, lymph node metastasis, distant metastasis, tumor grade, TNM stage and histological type; (3) operation information: records of surgery which including surgery date and specific surgery method postoperative therapy; and (4) follow‐up information: cause of death, cancer‐specific death (CSS). TNM stages were classified according to the American Joint Committee on Cancer (AJCC) TNM Classification for Lung and Pleural Tumors (eighth edition).

### Pathological diagnosis and *ALK* gene detection

The *ALK* gene rearrangement information was taken from pathology reports. As previously reported,^6^ the *ALK* gene status was detected by a fluorescence real‐time polymerase chain reaction‐based detection kit (Amoy Diagnostics Co. Ltd.). In addition, we used fluorescence in situ hybridization (FISH) for retesting to ensure the accuracy of the results. The Cy3‐labeled ALK probe was constructed by RiboBio. Fluorescence signals were generated using a fluorescence in situ hybridization kit (RiboBio), and a Nikon A1 confocal laser scanning microscope was used to take images and identification.

### Statistical analysis

Statistical analyses were performed using IBM SPSS Statistics 22.0 (IBM Inc.) and R version 3.3.2 (R Foundation for Statistical Computing, Vienna, Austria). The R package included survival, rms and ggplot2. Statistical significance was set at a two‐sided *p*‐value < 0.05. Kaplan–Meier and log‐rank tests were used to construct and compare survival curves. To explore the prognostic impact of *ALK* gene mutation, based on whether there were mutations in the ALK gene, we split the patients into positive and negative groups. Additionally, clinical variables with a *p*‐value < 0.1 in the univariate analyses were included in the multivariate models.

## RESULTS

### Patient characteristics

The baseline clinicopathological features of all patients included in the current study are summarized in Table 1. Compared to the negative group, the members of the ALK‐positive group were younger (*p* = 0.048), with a median age of 55, while those with negative *ALK* mutations were 60, more likely to be female (*p* = 0.022), there were fewer smokers (*p* = 0.046), they had different pathological subtypes (*p* = 0.001), larger primary tumors (*p* = 0.018), were at a more advanced stage (*p* = 0.007) and N classification (*p* = 0.004). Specifically, the risk of lymph node metastasis in ALK‐positive patients is greater than in ALK‐negative patients (28.8% vs. 18.3%; *p* = 0.028). These different points in baseline of two groups may impact overall survival. The most common primary site of ALK‐positive primary lung adenocarcinoma was the lower lobe, and the proportion in the positive and negative groups was 47.5% versus 33.3%. Additionally, The T classification was similar in patients belonging to both positive and negative groups (*p* = 0.051).

**TABLE 1:** Baseline characteristics of ALK+ and ALK‐ patients

	ALK (+)	ALK (−)	*p*‐value
Total evaluated	80	3031	
Age (years)			0.048
Mean ± SD	54.9 ± 12.9	60.8 ± 10.9	
Sex			0.022
Male	25 (31.2%)	1338 (44.1%)	
Female	55 (68.8%)	1693 (55.9%)	
Smoking history			0.046
No	54 (67.5%)	1625 (53.6%)	
Yes	22 (27.5%)	1209 (39.9%)	
Unknown	4 (5%)	197 (6.5%)	
Localization of primary tumor			0.017
LUL	13 (16.3%)	762 (25.1%)	
LLL	13 (16.3%)	498 (16.4%)	
RUL	18 (22.5%)	936 (30.9%)	
RML	8 (10%)	231 (7.6%)	
RLL	25 (21.3%)	512 (16.9%)	
Other	3 (3.8%)	92 (3.0%)	
Tumor size (cm)			0.018
Mean ± SD	2.13 ± 1.29	1.79 ± 1.33	
Pathological T stage			0.051
T1	63 (78.8%)	1952 (64.4%)	
T2	15 (18.8%)	881 (29.0%)	
T3	1 (1.3%)	83 (2.7%)	
T4	1 (1.3%)	115 (3.8%)	
Pathological N stage			0.004
N0	57 (71.2%)	2475 (81.7%)	
N1	8 (10.0%)	312 (10.3%)	
N2	14 (17.5%)	176 (5.8%)	
N3	1 (1.3%)	68 (2.3%)	
N+	23 (28.8%)	556 (18.3%)	
AJCC eighth stage			0.007
1	56 (70.0%)	2449 (80.8%)	
2	9 (11.3%)	267 (8.8%)	
3	15 (18.8%)	246 (8.1%)	
4	0 (0%)	69 (2.3%)	
Pathological type			0.001
Acinar predominant	60 (75%)	2155 (71.1%)	
Lepidic predominant	3 (3.8%)	149 (4.9%)	
Papillary predominant	7 (8.8%)	191 (6.3%)	
Micropapillary predominant	2 (2.5%)	31 (1.0%)	
Solid predominant	8 (10.0%)	170 (5.6%)	
Unknown	0 (0%)	335 (11.1%)	
Type of surgery			0.909
Lobectomy	51 (63.8%)	1973 (65.1%)	
Segmentectomy	9 (11.3%)	355 (11.7%)	
Wedge resection	20 (25.0%)	694 (22.9%)	
Others	0 (0%)	9 (0.3%)	
Targeted therapy			0.005
Yes	19 (23.8%)	367 (12.1%)	
No/unknown	61 (76.3%)	2664 (87.9%)	

### Survival analyses

**FIGURE 1:**
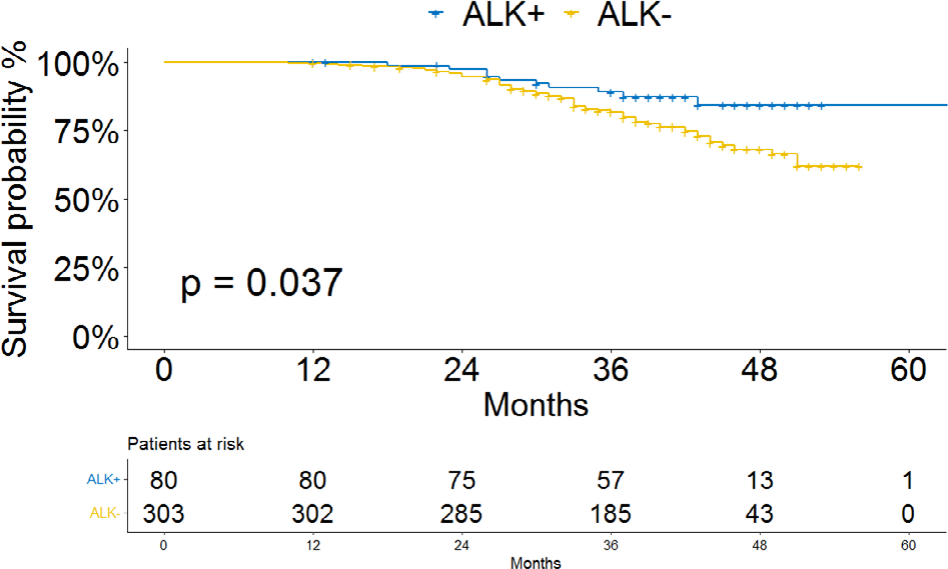
Survival analyses of surgically‐resected adenocarcinoma patients with ALK+ vs. ALK‐

After a 1:10 randomization, a total of 303 wild‐type patients were included in the survival analyses, with no significant differences in the baseline characteristics of wild‐type patients before and after sampling (Table 2). The survival curve for the two groups is shown in Figure 1. OS was better for ALK‐positive patients compared with ALK‐negative patients, The 3‐year cancer‐specific survival rates were 89.1 ± 3.7% versus 80.9 ± 2.3%, respectively (*p* = 0.037; Figure 1).

**TABLE 2:** ALK‐patient characteristics before and after 1:10 random sampling

	Before	After	*p*‐value
Total number of patients evaluated	3031	303	
Age (years)			0.856
Mean ± SD	60.8 ± 10.9	60.5 ± 9.2	
Sex			0.177
Male	1338 (44.1%)	146 (48.2%)	
Female	1693 (55.9%)	157 (51.8%)	
Smoking history			0.303
No	1625 (53.6%)	158 (52.2%)	
Yes	1209 (39.9%)	131 (43.2%)	
Unknown	197 (6.5%)	14 (4.6%)	
Localization of primary tumor			0.419
LUL	762 (25.1%)	75 (24.8%)	
LLL	498 (16.4%)	38 (12.5%)	
RUL	936 (30.9%)	92 (29.7%)	
RML	231 (7.6%)	17 (5.6%)	
RLL	512 (16.9%)	70 (23.1%)	
Other	92 (3.0%)	11 (3.6%)	
Tumor size (cm)			0.752
Mean ± SD	1.79 ± 1.33	1.75 ± 1.29	
Pathological T stage			0.152
T1	1952 (64.4%)	194 (64.0%)	
T2	881 (29.0%)	81 (26.8%)	
T3	83 (2.7%)	15 (4.9%)	
T4	115 (3.8%)	13 (4.3%)	
Pathological N stage			0.485
N0	2475 (81.7%)	242 (73.3%)	
N1	312 (10.3%)	36 (15.2%)	
N2	176 (5.8%)	21 (10.2%)	
N3	68 (2.3%)	4 (1.3%)	
N+	556 (18.3%)	81 (26.7%)	
AJCC eighth stage			0.611
1	2449 (80.8%)	249 (82.2%)	
2	267 (8.8%)	31 (10.2%)	
3	246 (8.1%)	27 (8.9%)	
4	69 (2.3%)	4 (1.3%)	
Pathological type			0.098
Acinar predominant	2155 (71.1%)	232 (76.6%)	
Lepidic predominant	149 (4.9%)	12 (4.0%)	
Papillary predominant	191 (6.3%)	16 (5.3%)	
Micropapillary predominant	31 (1.0%)	5 (1.7%)	
Solid predominant	170 (5.6%)	19 (6.3%)	
Unknown	335 (11.1%)	19 (6.3%)	
Type of surgery			0.379
Lobectomy	1973 (65.1%)	185 (6.1%)	
Segmentectomy	355 (11.7%)	38 (12.5%)	
Wedge resection	694 (22.9%)	78 (25.7%)	
Others	9 (0.3%)	2 (0.7%)	
Targeted therapy			0.429
Yes	367 (12.1%)	32 (10.6%)	
No/unknown	2664 (87.9%)	271 (89.4%)	

We also verified the correlation between survival and other factors. A total of 80 ALK‐positive lung adenocarcinoma participants were included in univariate and multivariate analyses to verify survival factors.

For ALK‐positive lung adenocarcinoma patients, univariate analyses revealed that age (*p* = 0.016), T stage (*p* = 0.008), N stage (*p* = 0.001), and AJCC stage (*p* < 0.001) were statistically significant predictors of tumor‐specific survival (Table 3). There was no significant difference in sex (*p* = 0.393), location (*p* = 0.293), primary site (*p* = 0.773), pathological subtype (*p* = 0.157), surgical method (*p* = 0.316) and targeted drug therapy (*p* = 0.334).

**TABLE 3:** Results of univariate and multivariate analyses of OS

	Univariate		Multivariate	
	*p*‐value	HR (95% CI)	*p*‐value	HR (95% CI)
Age				
≤60	Reference		Reference	
>60	0.016	6.724 (1.426, 31.691)	0.007	17.959 (2.217145.484)
Sex				
Male	Reference			
Female	0.393	1.966 (0.417, 9.271)		
Location				
Left	Reference			
Right	0.293	2.298 (0.487, 10.847)		
Lobe	0.773			
Upper lobe	Reference			
Middle lobe	0.816	0.775 (0.090, 6.636)		
Lower lobe	0.569	0.682 (0.183, 2.547)		
Other	0.985	NA		
Pathological type	0.157			
Acinar predominant	Reference			
Lepidic predominant	0.383	2.400 (0.336, 17.124)		
Papillary predominant	0.020	8.563 (1.412, 51.938)		
Micropapillary predominant	0.089	5.689 (0.765, 42.459)		
Solid predominant	0.709	1.590 (0.139, 18.208)		
Surgery type	0.316			
Lobectomy	Reference			
Segmentectomy	0.298	2.036 (0534, 7.765)		
Wedge resection	0.555	0.512 (0.055, 4.728)		
Pathological T stage	0.008		0.019	
T1	Reference		Reference	
T2	0.036	4.947 (1.106, 22.134)	0.053	5.800 (0.976, 34.450)
T3	0.003	15.155 (2.520, 91.134)	0.012	13.230 (1.769, 98.952
T4	0.005	29.127 (2.793,303.733)	0.006	89.827 (3.698,2181.751)
Pathological N stage	0.001		0.003	
N0	Reference		Reference	
N1	0.034	8.390 (1.174, 59.975)	0.008	20.530 (2.173,193.976)
N2	0.001	15.926 (3.198, 79.315)	0.001	21.070 (3.292,134.839)
AJCC eighth stage	<0.001			
1	Reference			
2	0.011	18.843 (1.957, 181.404)		
3	0.043	30.753 (3.689, 256.389)		
Targeted therapy				
No	Reference			
Yes	0.334	1.952 (0.503, 7.576)		
Targeted therapy (Patients after excluding stage I)				
No	Reference			
Yes	0.026	0.159 (0.032, 0.801)		

*Note*: NA: Impossible to calculate the specific value due to small sample size and no deceased patients.

According to multivariate analysis, age (*p* = 0.007), T stage (*p* = 0.019), and N stage (*p* = 0.003) remained independent prognostic predictors for ALK‐positive patients. The AJCC stage was not included in the multivariate analysis because it was not independent of T, N, and M stages. The details of the correlations between survival outcomes and parameters are shown in Table 3.

**FIGURE 2:**
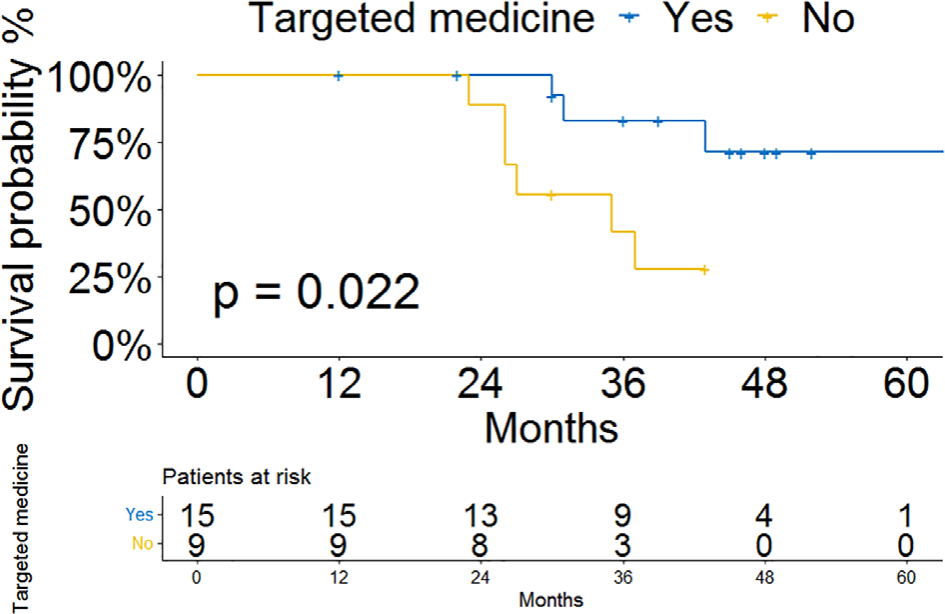
Survival analysis of surgically resected ALK rearranged patients (stage I patients were excluded) ‐ treated with vs without targeted medicine

It is worth noting that after excluding stage 1 patients, targeted drug therapy becomes a significant predictor of tumor‐specific survival (HR = 0.159; 95% CI: 0.032–0.801, *p* = 0.026). For advanced surgically‐resected ALK rearranged patients, the different groups of patient survival outcomes are shown in Figure 2. Patients who were administered targeted drugs had better survival compared to those who did not (*p* = 0.022).

## DISCUSSION

In the current study, we analyzed the clinical characteristics and prognosis of 80 ALK rearranged lung cancer patients who underwent radical surgical treatment in our department between 2016 and 2019 with 3031 ALK‐negative lung adenocarcinoma patients as controls. We also compared whether the use of targeted drugs affected survival outcome of ALK rearranged lung adenocarcinoma patients. ALK rearrangement is clinically important. Previous studies have indicated that the characteristics of ALK‐positive lung adenocarcinoma are different from other lung adenocarcinoma.^7^ *ALK* gene mutations also have reference significance for the prognosis of lung cancer.^8^ According to our previous study, the proportion of *ALK* gene mutation in surgically resectable lung adenocarcinoma (EML4‐ALK fusion and other ALK rearrangements) is 1.8%,^6^ a result consistent with the outcomes of other studies showing that *ALK* gene mutation accounts for 1.3%–7.9%^6^, ^9^ of patients with lung adenocarcinoma, depending on the population and methods of detection. Our research indicated the proportion of female patients is 68.8%, occupies the majority of *ALK* gene mutation patients, and in previous studies ranged from 51.2% to 67.9%.^10^ Moreover, the *ALK* mutation patients were more likely to be younger, which is consistent with some previous research results.^11^ Interestingly, several other ALK‐positive tumor patients are often younger, such as those patients with neuroblastomas, and inflammatory myofibroblastic tumors which occur most often in children and adolescents.^11^, ^12^

In addition, our research also showed that few *ALK* gene mutation patients had a smoking history and such findings are consistent with previous studies.^8^ Moreover, ALK rearranged patients had a higher risk of lymph node metastasis and more advanced stage, which had also been confirmed in other studies. Paik et al. also reported ALK‐positive lung adenocarcinoma seemed to be more likely to have lymph node metastasis.^12^ There are differences in baseline reports of lung adenocarcinomas with ALK mutations. Some parameters, such as racially diverse, detection methods and the data analysis may cause these differences.

Based on published studies for patients with lung adenocarcinoma who have undergone surgery, the prognostic value of ALK rearrangement in early‐stage lung adenocarcinoma is controversial. *ALK* mutation was viewed as an independent favorable prognostic predictor for OS according to our study; ALK rearranged patients had a better prognosis than other lung adenocarcinoma cases. Some previous studies have reported similar conclusions. The Lungscape project reported that ALK rearrangement is a favorable factor in resected lung adenocarcinoma patients,^5^ and a meta‐analysis also predicts better prognosis in NSCLC patients.^13^

Conversely, some studies have reported completely different conclusions. Gao et al. reported being ALK‐positive correlated with a poor prognosis and is an independent prognostic factor for predicting poor disease‐free survival (DFS) and OS.^14^ In addition, Yang et al. reported ALK positivity is associated with a significantly poor outcome in nonsmoking‐related lung cancer compared with ALK‐negative disease.^15^ The above study suggests a worse prognosis of ALK‐positive early‐stage (surgically resectable) patients may be associated with more aggressive characteristics of ALK‐positive LUAD. Some studies have shown that in ALK rearranged lung cancer patients, the proportion of advanced tumors and early lymph node metastasis is higher.^16^ Shin et al. also reported that patients with tumors with ALK rearrangement are more likely to develop lymph nodes metastasis, especially in early lung adenocarcinoma.^17^ Nevertheless, Paik et al. stated that ALK rearrangement did not affect the survival of lung cancer patients.^12^

There might be multiple reasons for the different effects of ALK fusion on the prognosis of lung cancer. First, previous studies included heterogeneous populations, and the results of research in different races may be different. Second, due to the low probability of *ALK* mutation, the number of patients included in each study was relatively small; some were even less than 30 cases, which may cause accidental errors. Third, different treatment methods may have affected the prognosis of the disease, For instance, targeted therapy may significantly improve the survival of ALK‐positive patients.

In our study, we found that for advanced ALK‐positive patients, targeted therapy was an independent better predictive factor of the prognosis (HR = 0.159; 95% CI: 0.032–0.801, *p* = 0.026). Targeted drugs, such as crizotinib and aletinib can effectively improve the survival of advanced patients. So we speculated that our study indicated a better prognosis for ALK‐positive patients might be related to the use of targeted drugs.

Targeted therapy has greatly improved the prognosis of patients with lung adenocarcinoma. For example, the development of targeted drugs for NSCLC with *EGFR* mutations has developed rapidly and is widely used clinically. Many randomized, phase 3 studies have shown that for EGFR‐positive lung cancer patients, targeted drugs, such as gefitinib and osimertinib, can significantly prolong disease‐free survival and improve prognosis in patients.^18^

Similarly, targeted drugs for ALK mutations have also shown good efficacy. A recent randomized, multicentre, open‐label, phase III study (NCT01828099) compared ceritinib and chemotherapy in stage IIIB/IV ALK‐rearranged NSCLC patients, which showed that ceritinib treatment significantly prolongs PFS. Median progression‐free survival was 16.6 months (95% CI: 12.6–27.2) in the ceritinib group versus 8.1 months (95% CI: 5.8–11.1) in the chemotherapy group (HR = 0.55; 95% CI: 0.42–0.73, *p* < 0.00001).^19^ Another randomized, controlled, open‐label, phase 3 trial (NCT01828112) for patients with ALK‐rearranged stage IIIB or IV NSCLC who had received previous chemotherapy and crizotinib but had disease progression also indicated that compared with chemotherapy, ceritinib significantly improved their prognosis.^20^ This indicates that even if crizotinib treatment fails, ALK‐positive patients can benefit from more effective ALK inhibitors.

Even though most patients with ALK rearranged lung adenocarcinoma have obtained benefits from TKIs, such as crizotinib, the long‐term prognosis may not be as satisfactory due to the emergence of acquired resistance. Many mechanisms have been identified since targeted drugs became widely used for clinical purposes.^21^ First, ALK‐dependent resistance has occurred, such as secondary mutations in the ALK tyrosine kinase domain. Numerous studies have shown that drug resistance can arise from reinducing kinase activation and signaling caused by secondary mutations. For example, in 2010, the first ALK resistance mutation ALK‐L1196M was found in crizotinib‐resistant patients^22^; such mutation modifies the ATP‐binding pocket and hinders TKI binding, and developed resistance to crizotinib. Subsequently, other mutations, such as G1269A, L1152R, and G1202R have also been reported. Although the frequency of ALK amplification is lower than that of secondary mutations, many studies have shown that the amplification of ALK is still a recognized cause of acquired resistance to crizotinib.^23^ Another resistance mechanism is ALK‐independent; in other words, activation of bypass signaling pathways, such as activation of EGFR was considered as the mechanism of resistance to crizotinib.^24^

The present study had several deficiencies which are worth mentioning. First, due to the relatively small sample size, the specific types of *ALK* gene mutations and the specific use of targeted drugs were not considered, which may influence patient outcome. Second, although the current study included a large number of ALK‐positive adenocarcinoma patients, we excluded advanced patients with inoperable tumors. Third, in this analysis, we focused only on survival time of patients without considering quality of life, which may not perfectly reflect patient survival. Lastly, this was a retrospective study and it would be much better to start a prospective study and see the long‐term results in ALK‐positive patients and their survival with targeted therapy.

In this study, we fully analyzed the clinical features and prognosis of ALK‐positive lung adenocarcinoma patients, and the effect of ALK inhibitors on the prognosis of ALK‐positive postoperative lung adenocarcinoma patients based on clinical data. We hope that our research can be verified in larger studies in the future and ultimately improve the clinical treatment of patients with ALK‐positive lung adenocarcinoma.

In conclusion, ALK rearrangement was an independent favorable prognostic predictor for OS in patients with completely surgically‐resected lung adenocarcinoma. Patients with ALK‐positive completely surgically‐resected lung adenocarcinoma have unique clinical features compared with other lung adenocarcinoma patients, including younger age, less are smokers, higher tumor stage and higher nodal stages. In our study, age, T stage, and N stage were independently associated with OS. Other than stage I, patients with surgically‐resected ALK‐positive lung adenocarcinoma may benefit from targeted therapy.

## CONFLICT OF INTEREST

The authors declare no competing interests in this work.
